# Dual Roles of IFN-γ and IL-4 in the Natural History of Murine Autoimmune Cholangitis: IL-30 and Implications for Precision Medicine

**DOI:** 10.1038/srep34884

**Published:** 2016-10-10

**Authors:** Bi-Jhen Syu, Chia-En Loh, Yu-Hsin Hsueh, M. Eric Gershwin, Ya-Hui Chuang

**Affiliations:** 1 Department of Clinical Laboratory Sciences and Medical Biotechnology, College of Medicine, National Taiwan University, Taipei, Taiwan; 2 Division of Rheumatology, Allergy and Clinical Immunology, University of California at Davis School of Medicine, Davis, CA 95616, USA

## Abstract

Primary biliary cirrhosis (PBC) is a progressive autoimmune liver disease with a long natural history. The pathogenesis of PBC is thought to be orchestrated by Th1 and/or Th17. In this study, we investigated the role of CD4^+^ helper T subsets and their cytokines on PBC using our previous established murine model of 2-OA-OVA immunization. We prepared adeno-associated virus (AAV)-IFN-γ and AAV-IL-4 and studied their individual influences on the natural history of autoimmune cholangitis in this model. Administration of IFN-γ significantly promotes recruitment and lymphocyte activation in the earliest phases of autoimmune cholangitis but subsequently leads to downregulation of chronic inflammation through induction of the immunosuppressive molecule IL-30. In contrast, the administration of IL-4 does not alter the initiation of autoimmune cholangitis, but does contribute to the exacerbation of chronic liver inflammation and fibrosis. Thus Th1 cells and IFN-γ are the dominant contributors in the initiation phase of this model but clearly may have different effects as the disease progress. In conclusion, better understanding of the mechanisms by which helper T cells function in the natural history of cholangitis is essential and illustrates that precision medicine may be needed for patients with PBC at various stages of their disease process.

Primary biliary cholangitis (PBC), hitherto called primary biliary cirrhosis, is considered a Th1 or Th17 disease with significant increases in IFN-γ and IL-17^1^,^2^,^3^,^4^,^5^,^6^. Within the liver, there is a significantly higher frequency of IFN-γ mRNA-positive cells^1^. In addition, there is an increased frequency of IL-17^+^ liver infiltrating lymphocytes^3^,^5^. Two independent genome-wide association studies have demonstrated that Th1 related IL-12A and IL-12RB2 variants are strongly associated with PBC^7^,^8^. Collectively, the data suggests an important role of CD4^+^ T helper subsets and their cytokines in disease pathogenesis. However, PBC has a long natural history and it is logical to consider that different mechanisms are in play at different stages of disease.

Our laboratory has attempted to define the natural history of biliary lesions by focusing on a molecular mimicry murine model of PBC induced following immunization with a mimotope of the highly conserved inner lipoyl domain of PDC-E2, the major mitochondrial autoantigen. Essentially mice immunized with either of two mimotopes, 2-octynoic acid-BSA (2-OA-BSA) or 2-octynoic acid-OVA (2-OA-OVA) develop high titer anti-mitochondrial antibodies (AMAs) and autoimmune cholangitis with lymphocytic infiltrates, portal inflammation, granuloma formation, and bile duct damage. In addition, if mice are stimulated with α-galactosylceramide (α-GalCer), which activates iNKT cells, the biliary damage is exacerbated and includes the appearance of fibrosis^9^,^10^,^11^,^12^. We report herein that administration of IFN-γ significantly promotes recruitment and lymphocyte activation in the earliest phases of autoimmune cholangitis but subsequently leads to downregulation of chronic inflammation through induction of the immunosuppressive molecule IL-30. In contrast, the administration of IL-4 does not alter the initiation of autoimmune cholangitis, but does contribute to the exacerbation of chronic liver inflammation and fibrosis. Better understanding of the mechanisms by which helper T cells function in the natural history of cholangitis is essential and illustrates that precision medicine, i.e. different therapies, may be needed for patients with PBC at various stages of their disease process.

## Results

### The cytokine profiles of 2-OA-OVA immunized model

**Figure 1:**
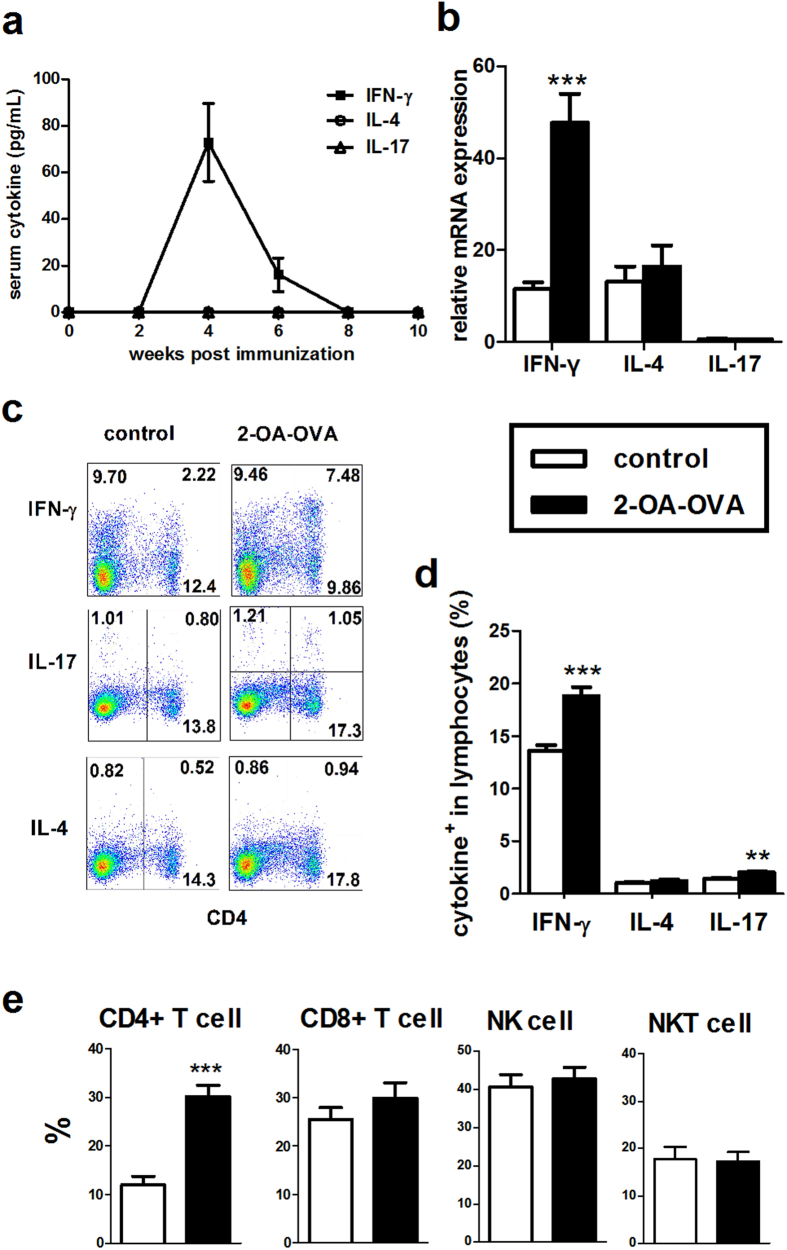
Cytokine profiles in serum and liver of mice immunized with 2-OA-OVA. Mice were immunized with 2-OA-OVA as described in the Material and Methods. (**a**) Serum levels of IFN-γ, IL-4, and IL-17 were detected at different time points post 2-OA-OVA immunization by ELISA. (**b–e**) Mice were sacrificed at 5 weeks and liver specimens were prepared individually. (**b**) Liver IFN-γ, IL-4, and IL-17 expressions were measured by qRT-PCR analysis. Cytokine expressions were normalized to β-actin and relative expression levels were shown. (**c,d**) The expressions of IFN-γ, IL-4 and IL-17 in liver lymphocytes were measured by flowcytometry. (**e**) The percentages of IFN-γ^+^ in CD4^+^ T, CD8^+^ T, NK (CD3^−^NK1.1^+^), and NKT (CD3^+^NK1.1^+^) cells were detected by flowcytometry. Control indicates results from mice immunized with adjuvant while without 2-OA-OVA. n = 8–11 mice per group. **p < 0.01; ***p < 0.001.

To understand the roles of Th1/IFN-γ and Th17/IL-17 in the pathogenesis of autoimmune cholangitis PBC, we first determined basal expression levels of IFN-γ, IL-4, and IL-17 in 2-OA-OVA immunized mice. As shown in Fig. 1a, IFN-γ was readily detected in serum and peaked at approximately 4 weeks post 2-OA-OVA immunization, while neither IL-17 nor IL-4 was detectable. There were no detectable levels of IFN-γ, IL-4 and IL-17 at any time points in control mice immunized with adjuvant alone (data not shown). IFN-γ mRNA expression was significantly increased in the liver of mice immunized with 2-OA-OVA, while neither IL-17 nor IL-4 was increased (Fig. 1b). We then determined cytokine expression in hepatic lymphocytes by stimulating cells with phorbol-myristate acetate (PMA) and ionomycin; 2-OA-OVA immunized mice had a higher frequency of IFN-γ expressing lymphocytes compared to the control group immunized with adjuvant without 2-OA-OVA, an increased frequency of IL-17 expressing lymphocytes was also noted, but the frequency was low in both groups. The percentage of IL-4^+^ in hepatic lymphocytes of both groups was also low (Fig. 1c,d). Strikingly, there was a markedly elevated frequency of IFN-γ expressing CD4^+^ T cells (Th1 cells) in 2-OA-OVA immunized mice, but no differences in the percentage of IFN-γ expression in hepatic CD8^+^ T, NK (CD3^−^ NK1.1^+^), and NKT (CD3^+^ NK1.1^+^) cells (Fig. 1e). These results suggested that the IFN-γ and Th1 mediated immune response was dominant in 2-OA-OVA immunized mice.

### Increased liver inflammation in AAV-IFN-γ treated 2-OA-OVA immunized mice

**Figure 2:**
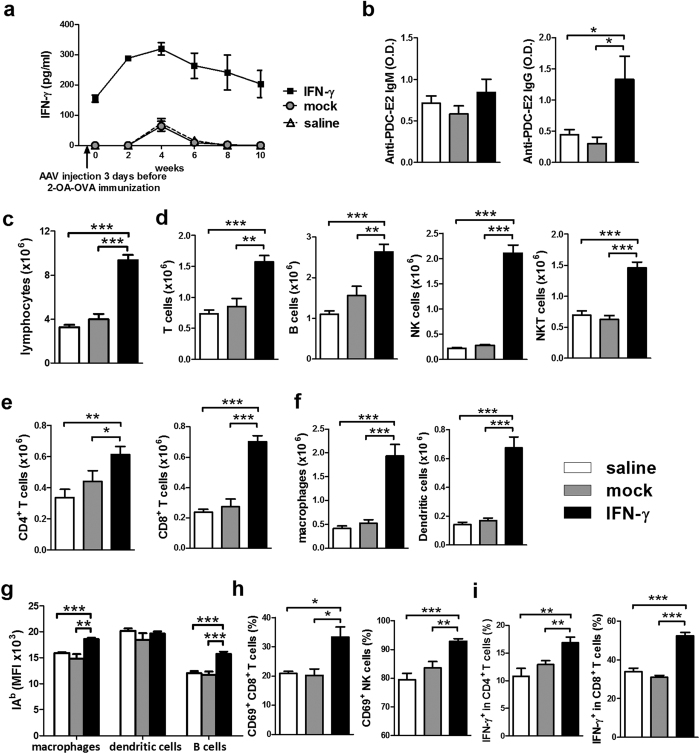
Increased AMA IgG and liver inflammation in AAV-IFN-γ treated 2-OA-OVA immunized mice. Mice were injected with AAV-IFN-γ, AAV mock or normal saline three days before the first 2-OA-OVA immunization. (**a**) Serum levels of IFN-γ were detected by ELISA. (**b–i**) Mice were sacrificed at 5 weeks and sera and liver specimens were prepared individually. (**b**) Serum levels of anti-PDC-E2 IgM and IgG were measured by ELISA. IgM, 1:150 dilution. IgG 1:300 dilution. O.D., optical density. (**c**) Liver lymphocytes were counted. (**d**) The numbers of T, B, NK, and NKT cells in the liver were measured. (**e**) The numbers of CD4^+^ and CD8^+^ T cells in the liver were measured. (**f**) The numbers of macrophages and dendritic cells in the livers were measured. (**g**) IA^b^ (MHC class II) expressions in macrophages, dendritic cells, and B cells were measured by flowcytometry. MFI, mean fluorescence intensity. (**h**) The percentages of CD69^+^ in CD8^+^ T cells and NK cells were measured. (**i**) The percentages of IFN-γ^+^ in CD4^+^ and CD8^+^ T cells were determined. n = 10–11 mice per group. *p < 0.05; **p < 0.01; ***p < 0.001.

To investigate the mechanism of how IFN-γ affected autoimmune cholangitis, we administered AAV-IFN-γ before 2-OA-OVA immunization. First, we examined mice for serum IFN-γ following treatment with a single intravenous injection of AAV- IFN-γ or mock virus and then immunized with 2-OA-OVA. As shown in Fig. 2a, IFN-γ was highly expressed in the serum of AAV- IFN-γ injected mice at all time points but basal expression only levels of IFN-γ were noted in controls injected with either 2-OA-OVA and mock virus or normal saline (Fig. 2a). At 5 weeks post 2-OA-OVA immunization, there was a significant increase in the titers of anti-PDC-E2 in AAV-IFN-γ treated 2-OA-OVA immunized mice (Fig. 2b). Total liver lymphocytes were increased 2.5-fold in AAV-IFN-γ treated 2-OA-OVA immunized mice (Fig. 2c). Lymphocyte subsets including T, B, NK, and NKT cells were all significantly increased. There was approximately a two-fold increase of T, B and NKT cells, and a 10-fold increase of NK cells in mice treated with IFN-γ (Fig. 2d). There was a 1.3 fold increase in CD4^+^ T cells but a 3-fold increase of CD8^+^ T cells (Fig. 2e). Of note, the numbers of professional antigen presenting cells, including B cells, macrophages and dendritic cells, in the liver were all significantly increased in AAV-IFN-γ treated 2-OA-OVA immunized mice (Fig. 2d,f). The expression of MHC class II on macrophages and B cells were also significantly increased (Fig. 2g). The frequencies of activated CD8^+^ T cells and NK cells were increased (Fig. 2h). In addition, the frequencies of IFN-γ secreting CD4^+^ and CD8^+^ T cells were significantly increased in AAV-IFN-γ treated 2-OA-OVA immunized mice (Fig. 2i).

**Figure 3:**
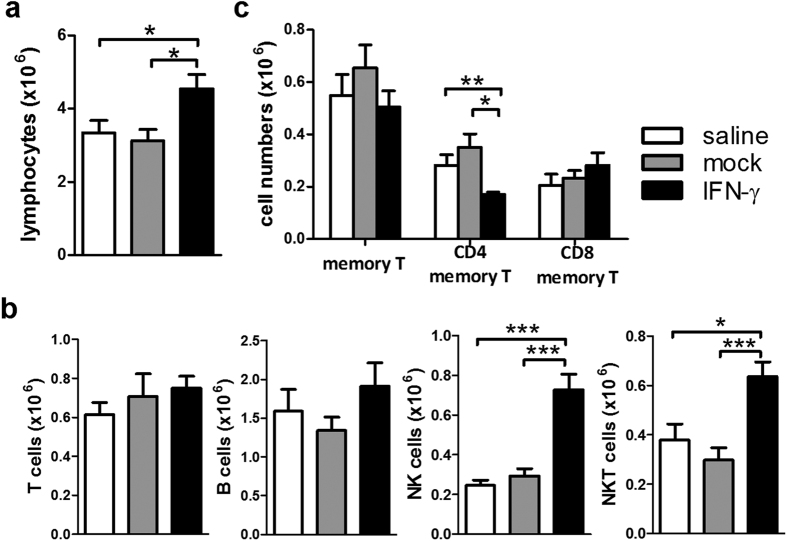
Increased NK and NKT cells at 10 weeks of AAV-IFN-γ treated 2-OA-OVA immunized mice. Mice were injected with AAV- IFN-γ, AAV mock or normal saline three days before the first 2-OA-OVA immunization and sacrificed at week 10. (**a**) Liver lymphocytes were counted. (**b**) The numbers of T, B, NK, and NKT cells in the liver were measured. (**c**) The numbers of memory T cells (CD3^+^ CD4^+^ CD44^+^ CD62L^−^ or CD3^+^CD8^+^CD44^+^ CD62L^−^) were measured. n = 10–11 mice per group. *p < 0.05; **p < 0.01; ***p < 0.001.

**Figure 4:**
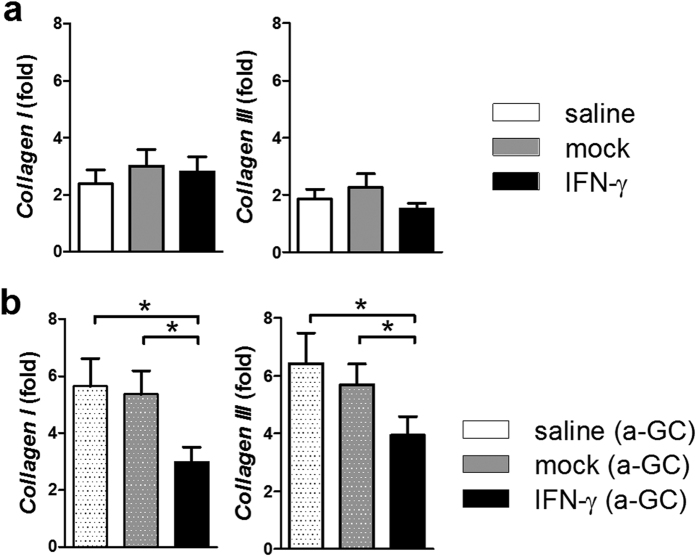
Decreased collagen levels in AAV-IFN-γ treated 2-OA-OVA immunized mice. (**a**) Mice were injected with AAV- IFN-γ, AAV mock or normal saline three days before the first 2-OA-OVA immunization and sacrificed at week 10. The expressions of collagen I and collagen III mRNA in the liver were detected by qRT-PCR. (**b**) Mice were injected with AAV- IFN-γ, AAV mock or normal saline three days before the first 2-OA-OVA/α-GalCer immunization and sacrificed at week 10. The expressions of collagen I and collagen III mRNA in the liver were detected by qRT-PCR. n = 10–15 mice per group. *p < 0.05.

At 10 weeks post 2-OA-OVA immunization, an increase in lymphocytic infiltrate in the AAV-IFN-γ treated 2-OA-OVA immunized mice could still be found. However, compared with a 2.5 fold increase at 5 weeks, there was only a 1.4 fold increase at 10 weeks (Fig. 3a). Threefold increase of NK cells and 1.5 fold increase of NKT cells were observed following IFN-γ administration. Neither T cells nor B cells were increased after 10 weeks of IFN-γ administration (Fig. 3b). However, the numbers of CD4^+^ memory T cells were decreased in the AAV-IFN-γ treated 2-OA-OVA immunized mice (Fig. 3c). As expected control mice immunized with 2-OA-OVA without α-GalCer as well as treated with AAV-mock and normal saline induced a slight increase of expression of collagen I and III, there were no changes in the expressions of collagen I and III in IFN-γ treatment (Fig. 4a). However, mice immunized with 2-OA-OVA and stimulated with α-GalCer had an approximate 6-fold expression of collagen I and III compared to naïve mice. 2-OA-OVA/α-GalCer immunized mice treated with AAV-IFN-γ had a lower expression of collagen I and III than controls (Fig. 4b). These results suggest that IFN-γ increased liver inflammation but subsequently led to reduced fibrosis.

### IL-30 expression in the liver correlated with IFN-γ expression in the liver

**Figure 5:**
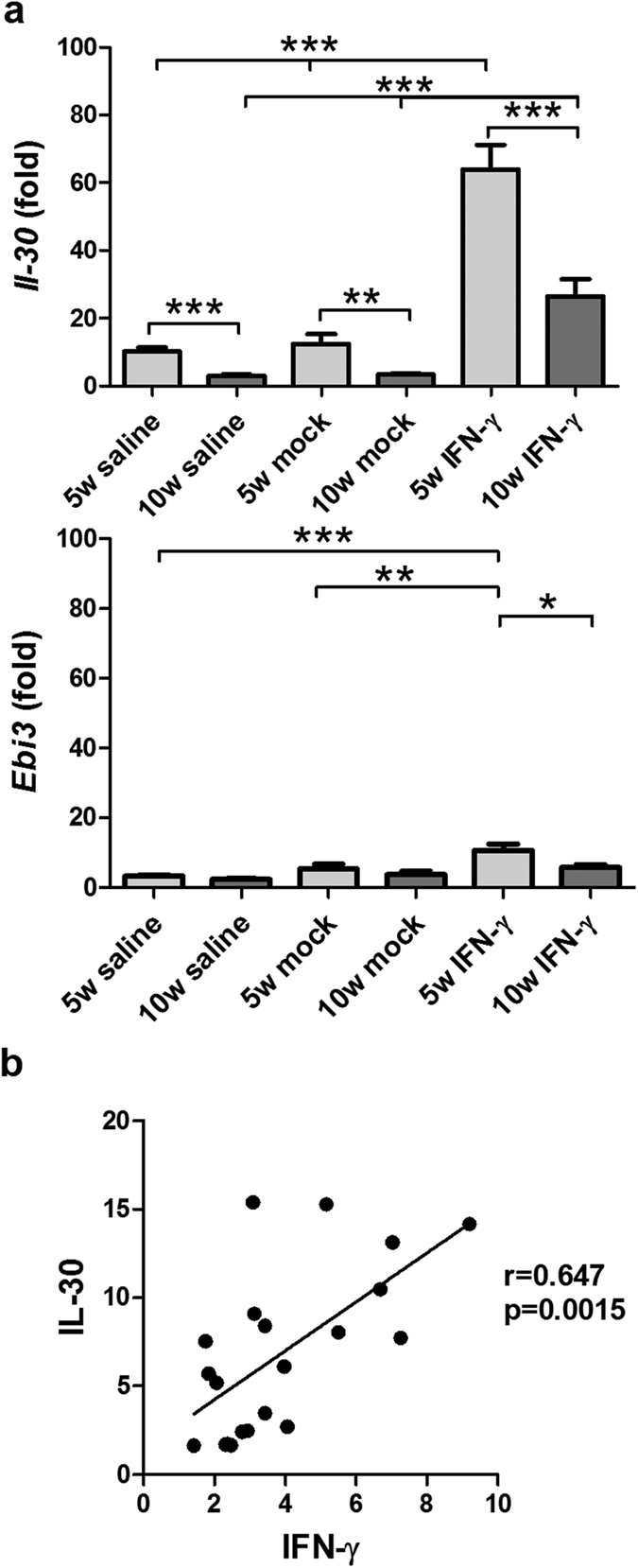
Increased IL-30 (IL-27 p28) in the liver of AAV-IFN-γ treated 2-OA-OVA immunized mice. Mice were injected with AAV- IFN-γ, AAV mock or normal saline three days before the first 2-OA-OVA immunization and sacrificed at weeks 5 and 10. (**a**) The expressions of IL-30 and EBi3 in liver were measured by qRT-PCR. This data represents the fold change of data normalized to the naïve mice. n = 10–11 mice per group. **p < 0.01; ***p < 0.001. (**b**) The relationship between the liver expression of IFN-γ and IL-30 in the normal saline treated 2-OA-OVA immunized mice. Each dot represents an individual mouse.

IFN-γ induces IL-30 expression in macrophages and dendritic cells^13^,^14^ and IL-30 inhibits IL-12-, IFN-γ-, and Con A- mediated hepatotoxicity by suppression of endogenous IFN-γ expression^13^. As shown in Fig. 5a, a 10-15-fold increase of IL-30 was found at 5 weeks post 2-OA-OVA immunization, while a 65-fold increase was noted in AAV-IFN-γ treated 2-OA-OVA immunization. However, there was only a 3-fold increase at 10 weeks post 2-OA-OVA immunization and a 26-fold increase in mice treated with AAV-IFN-γ. The expression level of the IL-27 subunit EBI3 was up-regulated in the liver with a similar but much lower pattern compared to p28 (IL-30) (Fig. 5a), suggesting that IL-30, not IL-27, was largely secreted following IFN-γ treatment. In addition, in 2-OA-OVA immunized mice, IL-30 expression in the liver significantly correlated with IFN-γ expression in the liver (r = 0.647, p < 0.01) (Fig. 5b). Taken together, IFN-γ induces IL-30 production which suppresses IFN-γ mediated inflammation.

### Administration of IL-4 did not reduce Th1 mediated autoimmune cholangitis but markedly exacerbated liver inflammation and fibrosis

**Figure 6:**
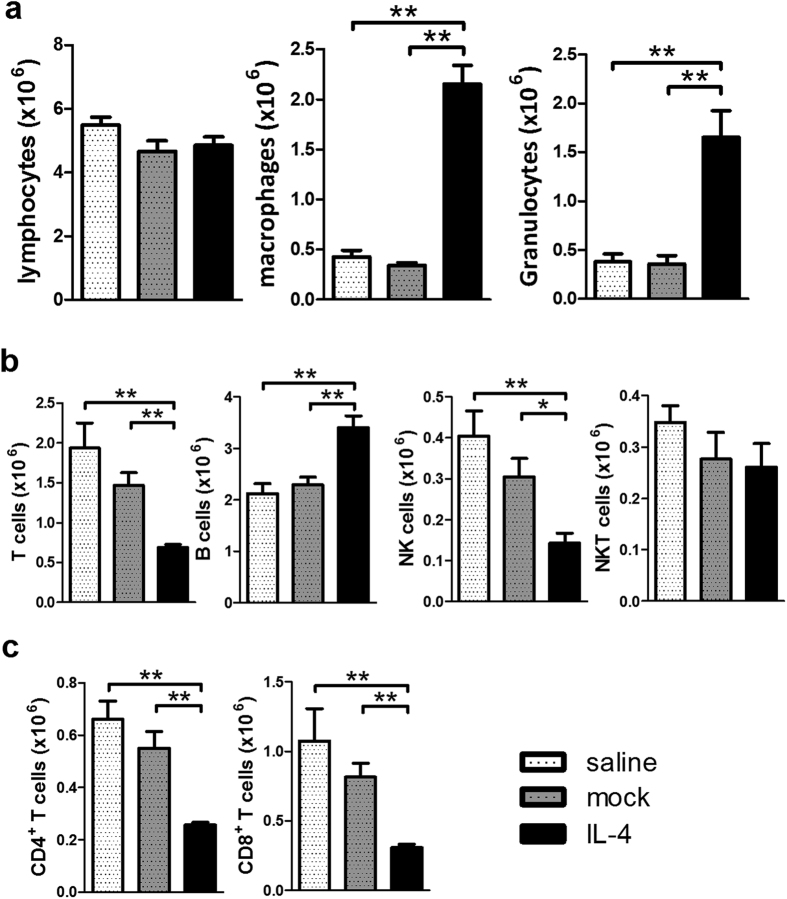
Increased macrophages and granulocytes but decreased T cells in AAV-IL-4 treated 2-OA-OVA/α-GalCer immunized mice. Mice were injected with AAV-IL-4, AAV mock or normal saline three days before the first 2-OA-OVA/α-GalCer immunization and sacrificed at week 5. (**a**) The numbers of lymphocytes, macrophages and granulocytes in the liver were measured. (**b**) The numbers of T, B, NK, and NKT cells in the liver were measured. (**c**) The numbers of CD4^+^ and CD8^+^ T cells in the liver were measured. n = 5 mice per group. *p < 0.05; **p < 0.01.

**Figure 7:**
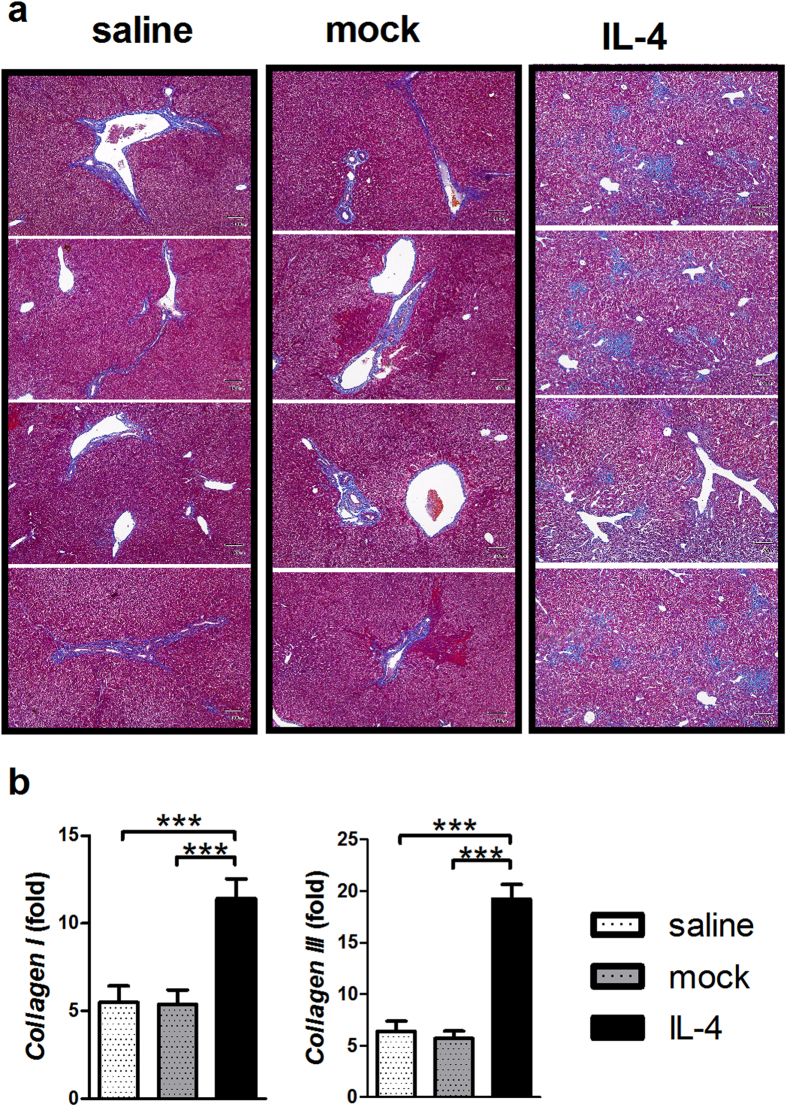
Augmented collagen levels in AAV-IL-4 treated 2-OA-OVA/α-GalCer immunized mice. Mice were injected with AAV-IL-4, AAV mock or normal saline three days before the first 2-OA-OVA/α-GalCer immunization and sacrificed at week 10. (**a**) Representative stained liver sections of Masson’s trichrome stain (x100 and magnification). The collagen fibers are stained blue. (**b**) The expressions of collagen I and collagen III mRNA in the liver were detected by qRT-PCR. n = 14–16 mice per group. ***p < 0.001.

We then investigated whether the Th2 cytokine IL-4 could suppress Th1 IFN-γ mediated autoimmune cholangitis and fibrosis. We treated mice as above with AAV-IL-4 and then immunized with 2-OA-OVA/α-GalCer using the protocol outlined above. At 5 weeks post immunization, there were no increases in liver lymphocyte infiltration (Fig. 6a). However, T cells, including CD4^+^ and CD8^+^ T cells, and NK cells were significantly decreased in IL-4 administered 2-OA-OVA/α-GalCer mice. In contrast, B cells in IL-4 administered 2-OA-OVA/α-GalCer mice were significantly increased (Fig. 6b,c). Further, we noted that the numbers of macrophages and granulocytes in the liver of IL-4 administered 2-OA-OVA/α-GalCer immunized mice were greatly increased compared to either the AAV mock or the normal saline control groups (Fig. 6a). Moreover, mice injected with AAV-IL-4 developed a significant induction of fibrosis 10 weeks following immunization (Fig. 7a). Consistent with this data, there were higher expressions of collagen I and III in mice treated with AAV-IL-4 compared to the AAV-mock or normal saline controls (Fig. 7b). These results suggest that administration of IL-4 decreased T cell infiltration but increased macrophages and granulocytes and exacerbated fibrosis.

**Figure 8:**
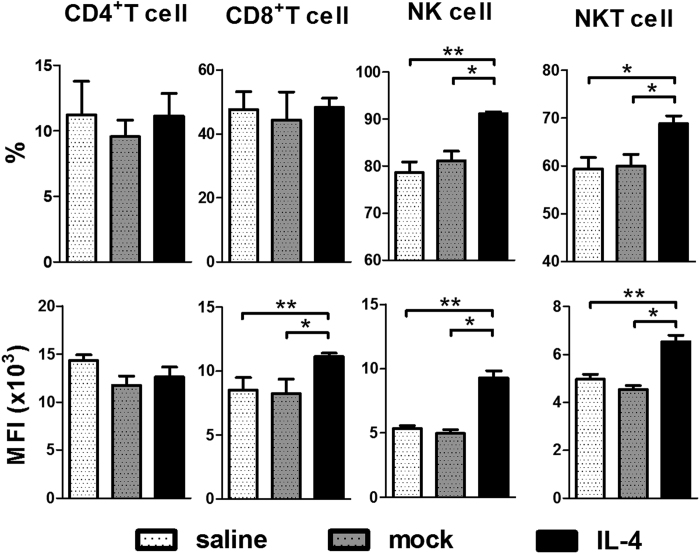
Increased IFN-γ production in CD8^+^ T, NK and NKT cells in AAV-IL-4 treated 2-OA-OVA/α -GalCer immunized mice. Mice were injected with AAV-IL-4, AAV mock or normal saline three days before the first 2-OA-OVA/α-GalCer immunization and sacrificed at week 5. The expression of IFN-γ in liver CD4^+^ T, CD8^+^ T, NK and NKT cells was measured by flowcytometry. MFI indicates mean fluorescence intensity of positive cells. n = 5 mice per group. *p < 0.05; **p < 0.01.

To investigate whether administration of IL-4 suppressed Th1 cells in this model, we analyzed the Th1 cells in liver at 5 weeks post 2-OA-OVA/α-GalCer immunization. Interestingly, we found that there were no differences in the frequency of IFN-γ producing CD4^+^ T cells and the expression level of IFN-γ in CD4^+^ T cells among three groups of 2-OA-OVA/α-GalCer immunized mice. Surprisingly, the expression level of IFN-γ in CD8^+^ T cells was increased in IL-4 injected mice. Furthermore, the frequencies and expression level of IFN-γ in NK and NKT cells of IL-4 administered 2-OA-OVA/α-GalCer mice were significantly higher than that of controls (Fig. 8). Hence, administration of IL-4 activates CD8^+^ T, NK and NKT cells to secrete more IFN-γ and exacerbate cholangitis.

## Discussion

The cellular immune response is critical in the natural history of pathology of PBC^3^,^4^,^5^,^15^,^16^,^17^,^18^,^19^,^20^,^21^. CD4^+^ T cells include at least three distinct subsets based on their cytokine production profile and function: Th1, Th2, and Th17. Th1 cells, characterized by the secretion of IFN-γ in response to IL-12, are a major defense in the eradication of intracellular pathogens, while Th2 cells, characterized by production of IL-4, IL-5 and IL-13, are activators of B cells for IgE production, eosinophil recruitment and mucosal expulsion mechanisms. Th17 cells, which secrete IL-17, IL-21, and IL-22, mediate host defensive mechanisms to various infections, especially extracellular bacterial infections^22^. The pathogenesis of organ-specific autoimmune diseases including multiple sclerosis, rheumatoid arthritis, type I diabetes, and Hashimoto’s thyroiditis are thought to be orchestrated and augmented by Th1 and/or Th17^23^,^24^. In contrast Th2 cells may exert down regulation^25^,^26^,^27^,^28^,^29^.

Autoreactive CD4^+^ T cells have been linked to the pathogenesis of PBC. Th1 cells and their signature cytokine, IFN-γ, and Th17 cells and IL-17 are thought to be the main pathogenic mediators in PBC^1^,^2^,^3^,^4^,^5^,^6^,^7^,^8^. In the 2-OA-OVA mimicry model, the numbers and levels of Th1 cells and IFN-γ are significantly increased and appear to correlate with the onset of disease^30^. Overexpression of IFN-γ enhances liver inflammation by increasing immune cell infiltrates, upregulating MHC class II expression of antigen presenting cells, promoting anti-PDC-E2 production, and activating CD4^+^ and CD8^+^ T and NK cells in initiation of disease. However, IFN-γ also protects the portal inflammation through IFN-γ-IL-30 axis at the effector phase. In contrast, IL-17 is not considered critical in this model; there are low levels of IL-17 in serum and liver of 2-OA-OVA immunized mice.

IFN-γ expression is readily detected in the serum and liver of patients with PBC^1^,^2^,^6^. Genome-wide association studies reflect that Th1 related IL-12A and IL-12RB2 variants are strongly associated with PBC^7^,^8^ and previous studies have demonstrated that deletion of IFN-γ in 2-OA-BSA-immunized mice reduced inflammatory portal infiltrates associated with prevention of bile duct damage^30^. In the study herein, we demonstrated that IFN-γ was significantly increased and IFN-γ enhanced liver inflammation in 2-OA-OVA immunization. In addition, we note that IL-12p40^−/−^ dnTGFβRII mice manifest a dramatic reduction in histological autoimmune cholangitis and significant decreases in levels of intrahepatic proinflammatory cytokines^31^. These results suggest that Th1 cells and IFN-γ are the dominant contributors in the initiation of the disease by increasing numerous immune cell infiltrates, upregulating MHC class II expression of antigen presenting cells, promoting anti-PDC-E2 antibodies production, and activating CD4^+^ and CD8^+^ T cells and NK cells. Indeed our data is consistent with recent data in another murine model that mice with a deletion of the IFN-γ 3’ untranslated region adenylate-uridylate–rich element have prolong and chronic expression of IFN-γ and develop a primary biliary cholangiopathy similar to PBC, which likewise proposes a key role of IFN-γ in disease initiation^32^,^33^.

IFN-γ is regarded traditionally as a critical proinflammatory mediator in several Th1-driven autoimmune disease model systems^23^. However, despite the expected overall proinflammatory and disease-enforcing role in some models, there is also evidence for protection against inflammation in other models^34^,^35^,^36^,^37^,^38^. The latter data is consistent our thesis that IFN-γ has a dual role in the natural history of an autoimmune disease, particularly a disease with a long natural history^39^,^40^. Our data also reveals a potential role of IL-30. IL-30, a subunit p28 of IL-27, has been detected in macrophages, dendritic cells, and hepatocytes^13^,^41^. Induction of high expression of IL-30 occurs with the coordination of activated T cells^42^. In liver injury, IL-30 suppresses the intrinsic ability of CD4^+^ T cells to produce IFN-γ in acute liver inflammation^13^,^41^ and IL-30 attenuates liver fibrosis by recruiting natural-killer–like T cells to the liver to remove activated hepatic stellate cells^43^. IL-30 has also been shown to inhibit central nervous system autoimmunity via antagonizing Th1 and Th17 responses in experimental autoimmune uveitis^44^,^45^. More studies are needed to understand how IL-30 inhibits autoimmune cholangitis but we submit that IFN-γ promotes disease by initially activating and recruiting immune cells and thence triggers robust immunosuppressive IL-30 production which reduces the liver inflammation.

Consistent with earlier data, mice immunized with 2-OA-OVA without α-GalCer did not induce collagen production and fibrosis while mice injected with 2-OA-OVA and α-GalCer manifest collagen production and fibrosis^9^,^10^. The administration of IFN-γ did not induce collagen production in 2-OA-OVA immunized mice. In fact, overexpression of IFN-γ decreases collagen production in 2-OA-OVA/α-GalCer immunized mice. α-GalCer is a well-defined potent and specific ligand for iNKT cell activation in both humans and mice. Upon ligation of iNKT cell receptors with α-GalCer, iNKT cells rapidly produce large amounts of cytokines, including IFN-γ and IL-4^11^.

IL-4 is not normally considered to play a major role in autoimmune disease, i.e. experimental autoimmune encephalomyelitis (EAE), multiple sclerosis (MS), rheumatoid arthritis (RA), inflammatory bowel disease (IBD), or psoriasis^46^,^47^,^48^. However, systemic IL-4 immunotherapy has been shown to improve some Th1/Th17-associated autoimmune diseases^25^,^26^,^27^,^28^,^29^. In our study, IL-4 levels in 2-OA-OVA immunized autoimmune cholangitis remained at basal values. Nonetheless the administration of IL-4 increased liver inflammation with marked increases in macrophage, granulocyte and IFN-γ secreting CD8^+^ T, NK and NKT cell populations. Gene transfer of IL-4 in the liver of naïve mice induces hepatitis characterized by hepatocyte apoptosis and a massive macrophage infiltrate^49^,^50^ and IL-4 administration in primates is associated with hepatic damage^51^,^52^. Overall our data demonstrates Th1 cells and IFN-γ are the dominant contributors in the initiation phase of this model but clearly may have different effects as the disease evolves PBC. Delineating the varying activities of IFN-γ during the course of disease provides insight into the complex role of IFN-γ in autoimmunity and illustrates the complexities of treating a disease like PBC with biologics and the concept that one size may not fit all patients.

## Methods

### Experimental mice

Female C57BL/6 mice aged 7–9 weeks were obtained from the National Laboratory Animal Center, Taiwan and mice maintained in the Animal Center of the College of Medicine, National Taiwan University. This study was approved by the Institutional Animal Care and Use Committee (IACUC) of National Taiwan University College of Medicine and College of Public Health, and performed in accordance with the approved guidelines.

### Preparation of AAV-IFN-γ and AAV-IL-4

cDNA containing murine IFN-γ or IL-4 were inserted into a recombinant adeno-associated viral vector (pAAV-IRES-GFP) (Cell Biolabs, San Diego, CA, USA). The cytokine genes were inserted using a pAAV-IRES-GFP plasmid which was co-transfected with pAAV-DJ and pHelper at a ratio of 1:1:1 into the adenovirus packaging AD293 cell line. Viruses were purified from infected cells 42–48 hours after infection by three freeze-thaw cycles followed by a Hi-Trap Heparin column. Viral titers (transduction unit, TU) were measured by GFP expression in infected 293T cells using flow cytometry. Throughout these studies a mock AAV was used as a control; it did not contain a transgene in the expression cassette. Details of the preparation of these viral vectors, including positive and negative controls, are described elsewhere^12^.

### Experimental protocol

Female C57BL/6 mice, at 7–9 weeks of age, were intraperitoneally immunized with 2-OA-OVA in the presence of complete Freund’s adjuvant (CFA, Sigma-Aldrich, St. Louis, MO, USA) and subsequently boosted at weeks 2, 4, 6 and 8 with 2-OA-OVA in incomplete Freund’s adjuvant (IFA, Sigma-Aldrich). Mice were sacrificed at 5 weeks post 2-OA-OVA immunization for determining of IFN-γ, IL-4 and IL-17 in liver tissues by quantitative real-time RT-PCR analysis and cytokine expressing immune cells by flow cytometry. In a subsequent set of experiments, and based on our data on the influence of α-GalCer on fibrosis in this model^9^,^10^,^11^,^12^, two μg of α-GalCer (Funakoshi, Tokyo, Japan) were injected with the first and second 2-OA-OVA immunizations. AAV-IFN-γ and AAV-IL-4 were administered to mice at 3 days before the first mimeotope immunization. Mice were sacrificed at 5 or 10 weeks post-immunization for liver histopathology, definition of mononuclear cell phenotypes, assay of IFN-γ production, and titers of AMAs.

### Isolation of mRNA and real-time PCR

Total RNA from liver specimens was obtained by the TRIzol method (Invitrogen Life Technologies, Carlsbad, CA, USA). The cDNA was generated by oligonucleotide priming using High-Capacity cDNA Reverse Transcription Kits (Applied Biosystems, Foster City, CA, USA). Amplification was performed with SYBR Green MasterMix (Thermo Scientific, USA) using the 7500 Real-Time PCR System (Applied Biosystems). Results were analyzed by 2^−ΔΔCt^ relative quantification method and normalized to β-actin.

### Liver mononuclear cell quantitation and cytokine detection

Livers were perfused with PBS containing 0.2% BSA (PBS/0.2% BSA), passed through a 100 μm nylon mesh, and re-suspended in PBS/0.2% BSA. The parenchymal cells were removed as pellets after centrifugation at 50 g for 5 minute and the non-parenchymal cells isolated using 40% and 70% Percoll (GE HealthCare Biosciences, Quebec, Canada). After centrifugation, collected cells were washed with PBS/0.2% BSA and the viability confirmed by trypan blue dye exclusion. Subsets of liver mononuclear cells were measured by flow cytometry. Before staining cells, with a previously defined optimal dilution of monoclonal antibodies (Abs), the cells were pre-incubated with anti-CD16/32 (clone 93) to block non-specific FcRγ binding. The following mAbs were used in this study: anti-CD3, anti-CD4, anti-CD8a, anti-CD19, anti-NK1.1, anti-CD69, anti-CD44, anti-CD62L, anti-IA^b^, anti-CD11b, anti-CD11c, anti-F4/80, anti-Gr1, anti-Ly6G (Biolegend, San Diego, CA, USA). For cytokine detection, liver mononuclear cells were stimulated with phorbol-myristate acetate (PMA,  1 ng/ml, Sigma-Aldrich) and ionomycin (1 μg/ml, Sigma-Aldrich) for 4 or 18 hours and IFN-γ, IL-4, or IL-17 intracellular staining was performed after cell surface staining. Stained cells were measured with a flow cytometer (BD Biosciences) and analyzed using FlowJo software (Tree Star, Inc., Ashland, OR, USA). Optimal concentrations of the mAbs were used throughout and all assays included positive and negative controls.

### Serum AMAs and cytokine detection

Serum titers of IgM and IgG anti-PDC-E2 autoantibodies were measured by ELISA using our well standardized recombinant mouse PDC-E2 as described previously^10^,^11^,^12^. Serum levels of IFN-γ, IL-4 and IL-17 were assayed by ELISA (R&D Systems, Minneapolis, MN, USA).

### Histopathology

Portions of liver were excised and immediately fixed with 10% buffered formalin solution for 2 days at room temperature. Paraffin-embedded tissue sections were then cut into 4-μm slices for Masson’s trichrome staining.

### Statistical analysis

Mann-Whitney U analysis was used to determine significant differences between groups. The Spearman test was used to evaluate correlations (Prism 5; Graph-Pad Software, La Jolla, CA, USA). Results are expressed as the mean ± standard error of the mean (SEM).

## Additional Information

**How to cite this article**: Syu, B.-J. *et al.* Dual Roles of IFN-γ and IL-4 in the Natural History of Murine Autoimmune Cholangitis: IL-30 and Implications for Precision Medicine. *Sci. Rep.* **6**, 34884; doi: 10.1038/srep34884 (2016).
